# Tryptogalinin Is a Tick Kunitz Serine Protease Inhibitor with a Unique Intrinsic Disorder

**DOI:** 10.1371/journal.pone.0062562

**Published:** 2013-05-03

**Authors:** James J. Valdés, Alexandra Schwarz, Israel Cabeza de Vaca, Eric Calvo, Joao H. F. Pedra, Victor Guallar, Michalis Kotsyfakis

**Affiliations:** 1 Institute of Parasitology, Biology Centre of the Academy of Sciences of the Czech Republic, České Budějovice, Czech Republic; 2 Joint Barcelona Supercomputing Center-Institute for Research in Biomedicine (BSC-IRB) Research Program in Computational Biology, Barcelona Supercomputing Center, Barcelona, Spain; 3 Institució Catalana de Recerca i Estudis Avançats (ICREA), Barcelona, Spain; 4 Vector Biology Section, Laboratory of Malaria and Vector Research, National Institute of Allergy and Infectious Diseases, National Institutes of Health, Rockville, Maryland, United States of America; 5 Center for Disease Vector Research and Department of Entomology, University of California Riverside, Riverside, California, United States of America; University of Minnesota, United States of America

## Abstract

**Background:**

A salivary proteome-transcriptome project on the hard tick *Ixodes scapularis* revealed that Kunitz peptides are the most abundant salivary proteins. Ticks use Kunitz peptides (among other salivary proteins) to combat host defense mechanisms and to obtain a blood meal. Most of these Kunitz peptides, however, remain functionally uncharacterized, thus limiting our knowledge about their biochemical interactions.

**Results:**

We discovered an unusual cysteine motif in a Kunitz peptide. This peptide inhibits several serine proteases with high affinity and was named tryptogalinin due to its high affinity for β-tryptase. Compared with other functionally described peptides from the Acari subclass, we showed that tryptogalinin is phylogenetically related to a Kunitz peptide from *Rhipicephalus appendiculatus*, also reported to have a high affinity for β-tryptase. Using homology-based modeling (and other protein prediction programs) we were able to model and explain the multifaceted function of tryptogalinin. The N-terminus of the modeled tryptogalinin is detached from the rest of the peptide and exhibits intrinsic disorder allowing an increased flexibility for its high affinity with its inhibiting partners (i.e., serine proteases).

**Conclusions:**

By incorporating experimental and computational methods our data not only describes the function of a Kunitz peptide from *Ixodes scapularis*, but also allows us to hypothesize about the molecular basis of this function at the atomic level.

## Introduction

The first Kunitz peptide was discovered by Moses Kunitz from bovine pancreas (BPTI) [1], and since then Kunitz peptides have been identified as a diverse protein family (from plants to animals), affecting different serine proteases with distinct binding affinities [2], [3]. There are several tick salivary Kunitz peptides described in the literature as potent protease inhibitors with unique and stringent target specificity. For instance, the tick anticoagulant peptide from *Ornithodoros moubata* is a potent inhibitor of factor Xa, but has no effect on factor VIIa, kallikrein, trypsin, chymotrypsin, thrombin, urokinase, plasmin, tissue plasminogen activator and elastase [4]. Another example is the tick-derived protease inhibitor (TdPI) from the hard tick *Rhipicephalus appendiculatus* that is a potent β-tryptase inhibitor, but not for urokinase, thrombin, factor Xa, factor XIIa, elastases, kallikreins, cathepsin G, granzyme B, chymase and chymotrypsins [5].

Hard tick feeding lasts up to a week as opposed to their distant relative, the soft ticks, whose feeding cycle is much faster [6]. Because of the extended hard tick feeding cycle, a complex of host defense responses takes place at the injury site that is counteracted by the pharmacological properties of tick saliva [6], [7], [8]. Tick salivary protease inhibitors play a role in regulating host proteolytic events [9] and the transmission of tick-borne diseases, such as Lyme disease [10], while other tick salivary proteins facilitate the transmission of rickettsioses [11] and tick-borne encephalitis [12]. Because of the known pharmacological properties of tick saliva (and the ability to facilitate tick-borne pathogen transmission), two salivary gland transcriptome and proteome projects – also called sialome projects – revealed secreted salivary proteins expressed from the hard tick, *Ixodes scapularis* [13], [14]. Annotating these sialome projects amounted to hundreds of tick salivary sequences that remain uncharacterized. These projects revealed many protein sequences classified as having the conserved Kunitz domain [13], [14] and 60 sequences are annotated as monolaris – sequences that have six cysteine (Cys) residues forming three disulfide bridges and a single Kunitz head [14]. These 60 monolaris sequences can be further divided into subgroups categorized by variations in their Cys motif. The remaining Kunitz sequences (a total of 32) from *I. scapularis* are defined as bilaris (two Kunitz heads) and penthalaris (five Kunitz heads).

In our study we focused on the most abundant Kunitz group from the *I. scapularis* sialome project by Ribeiro et al. [14]: the monolaris group. We identified a Kunitz sequence that displays an unusal Cys motif when compared with the other monolaris and to previously reported Kunitz peptides. Since tick Kunitz peptides are known to inhibit serine proteases we performed an inhibitory screening demonstrating that this *I. scapularis* Kunitz inhibits several proteases as well as being a potent inhibitor of human skin β-tryptase (HSTβ). Furthermore, a phylogenetic analysis using several functionally described Kunitz protease inhibitors from hematophagous arthropods, nematodes and platyhelminthes reveals that this *I. scapularis* Kunitz is closely related to TdPI. We will, hereafter, refer to this *I. scapularis* Kunitz as tryptogalinin due to its high affinity for HSTβ. Since the crystal structure of TdPI and its complex with trypsin has been solved, we used *in silico* methods to elucidate the biophysical principles that determine tryptogalinin’s protein fold, to predict its global tertiary structure and to hypothesize about its physicochemical interactions with serine proteases that account for its biochemical specificity – when compared with TdPI.

## Materials and Methods

### General Experimental Procedures

Unless otherwise indicated, standard procedures were followed according to Sambrook et al. [15]. Experiments were performed at room temperature (25±1°C). All water used was of 18-MΩ quality produced by a MilliQ apparatus (Millipore). If not otherwise stated, all reagents were purchased from Sigma-Aldrich.

### Peptide Expression

The experimental procedures for tryptogalinin (GenBank: DN971582) overexpression and purification were previously described in Chmelar et al. [7] with the exception that tryptogalinin overexpression was done in BL21(DE3)pLysE bacterial cells (Invitrogen).

### Serine Protease Inhibition Assays

All assays were performed at 30°C with a total of 340 nM of tryptogalinin that was pre-incubated with each enzyme for 10 min before adding the respective fluorescent substrate of the enzyme. A *t*-test was used to statistically analyze the observed inhibition of tryptogalinin with a statistical significance set at *p*≤0.05 when evaluating the enzymatic activity in the presence or absence of this inhibitor. For both assays, the hydrolysis rate of the fluorescent substrate was estimated using the slope from the linear fit (arbitrary fluorescence units per sec; *R^2^*≥0.95) of the data in triplicates (the mean substrate hydrolysis rate of the triplicates and the standard error of this mean were calculated). Substrate hydrolysis rate was followed in a Tecan Infinite M200 96-well plate fluorescence reader (Tecan group Ltd) using 365 nm excitation and 450 nm emission wavelengths with a cutoff at 435 nm. The linear fit of the fluorescence increase as a function of time was verified with the Magellan™ - Data Analysis Software (Tecan group Ltd). The observed substrate hydrolysis rate in the absence of protein was considered as 100% when compared with the remaining enzymatic activity in the presence of the protein.

All enzymes, except for trypsin (bovine), were of human origin, tissue-purified or recombinant. The following assay concentrations and enzymes were purchased from Sigma-Aldrich: thrombin (0.02 nM), α-chymotrypsin (0.06 nM), plasmin (0.25 nM), and chymase (4.2 nM). Human skin β-tryptase (0.01 nM) was purchased from Promega, factor Xa (1 nM) from EMD Biosciences, factor XIIa (1.8 nM) from Haematologic Technologies Inc., kallikrein (1.2 nM) from Fitzgerald Industries International, elastase (0.33 nM) from Elastin Products, Factor XIa (0.16 nM), urokinase Plasminogen Activator (uPA; 0.67 nM), and tissue Plasminogen Activator (t-PA; 0.06 nM) were purchased from Molecular Innovations. Matriptase (1 nM) was obtained from R&D Systems and sequencing-grade trypsin (0.25 nM) was purchased from Roche.

The following assay buffers were prepared. For elastase and chymase: 50 mM Hepes buffer, pH 7.4, 100 mM NaCl, 0.01% Triton X-100; for trypsin, α-chymotrypsin, factor Xia and factor XIIa: 50 mM Tris-HCl, pH 8, 150 mM NaCl, 20 mM CaCl_2_, 0.01% Triton X-100; for thrombin: 50 mM Tris-HCl, pH 8, 150 mM NaCl, 0.01% Triton X-100; for β-tryptase: 50 mM Tris-HCl, pH 8, 50 mM NaCl, 0.05% Triton X-100; for kallikrein, matriptase and plasmin: 20 mM Tris–HCl, pH 8.5, 150 mM NaCl, 0.02% Triton X-100; for factor Xa: 20 mM Tris-HCl, pH 8, 200 mM NaCl, 5 mM CaCl_2_, 0.1% BSA; and, for uPA and tPA: 20 mM Tris-HCl, pH 8.5, 0.05% Triton X-100. The substrates used were Suc-Ala-Ala-Pro-Val-AMC for elastase, Boc-Asp-Pro-Arg-AMC for thrombin and plasmin, Boc-Gln-Ala-Arg-AMC for trypsin, factor XIa and uPA (Sigma-Aldrich), Boc-Phe-Ser-Arg-AMC for β-tryptase, Suc-Leu-Leu-Val-Tyr-AMC for chymase (Bachem Bioscience, Inc.), Suc-Ala-Ala-Pro-Val-AMC for α-chymotrypsin (EMD Biosciences) and methylsulfonyl-D-cyclohexylalanyl-Gly-Arg-AMC acetate for factor Xa, factor XIIa, tPA, matriptase, and kallikrein (American Diagnostica Inc.). All substrates were at 250 µM final concentration for each assay.

To calculate the apparent *K_i_* (*K_i_*_(app)_) we used the BotDB server [16]. For an enzyme-substrate-inhibitor reaction the BotDB server calculates the *K_i_*_(app)_ based on the amount of enzyme and subsrate (S) used, the Michaelis constant (*K_m_*), and the inhibitory concentration that reduces 50% of the enzymatic activity (*IC50*). Based on the work by Cheng and Prusoff [17], the equation that the BotDB server uses to calculate the *K_i_*_(app)_ in a classical inhibitory reaction is: 
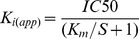



Due to its tetrameric nature, for the inhibition of HSTβ we adopted the method described by Schaschke et al. [18], by using a non-linear regression to calculate the *K_i_*_(app)_ and by correcting the competition between the inhibitor and the substrate.

### *I. scapularis* Monolaris Multiple Sequences Alignment

The monolaris nucleotide sequences found in *I. scapularis* sialome [14] were submitted to the NCBI Open Reading Frame Finder (ORF) online server (http://www.ncbi.nlm.nih.gov/gorf/gorf.html) to verify and edit the sequences to an appropriate start-stop codon. Accordingly, we only used sequences containing a start and stop codon and a signal peptide. The translated amino acid sequences that were provided by the ORF Finder were subsequently submitted to the SignalP 4.0 server [19] and the signal peptide was removed from all protein sequences. Only 4 sequences out of the 60 sequences, reported by Ribeiro et al. [14], were removed using the aforementioned screening. The remaining 56 *I. scapularis* monolaris sequences were included for primary sequence alignment using MAFFT version 7 [20].

### Sequence Alignment and Phylogenetic Analysis of Functionally Described Kunitz Peptides

For our phylogenetic analysis, we searched the literature using NCBI database and PubMed for secreted and functionally described Kunitz protease inhibitors from hematophagous arthropods, nematodes and platyhelminthes to depict the relationship of these inhibitors with the *I. scapularis* Kunitz peptide, tryptogalinin (GenBank: DN971582). Full-length amino acid sequences were submitted to the SignalP 4.0 server [19] and the signal peptide was removed from all protein sequences. All sequences were aligned by a homology alignment profiling strategy based on the published Kunitz peptide sequences that were retrieved from the SwissProt database via BLAST (Figure S2). We used the program MAFFT version 7 for this multiple sequence alignment with an iterative refinement method (L-INS-I) and the BLOSUM 62 matrix (Gap opening penalty: 1.5, Offset value: 0.1) [20]. Afterwards, unaligned, flanking sequence regions were excised and gaps produced in the alignment by a minimum of three sequences were closed. We used Prottest 3.0 to analyze the final alignment in order to select the best-fit amino acid substitution model for the phylogenetic reconstruction [21]. The phylogenetic Kunitz tree was constructed under the maximum likelihood (ML) optimality criterion and the WAG model [22], with a gamma distribution of substitution rate, using the program RAxML (HPC version 7.2.6) [23]. Tree searching and bootstrapping were performed simultaneously (-f a, 1000 bootstrap replicates). Hg1, a trypsin and potassium channel inhibitor of the Mexican scorpion *Hadrurus gertschi* (GenBank: P0C8W3) [24], was chosen as an outgroup in our phylogenetic reconstruction since scorpions represent the closely related sister group to the Acari subclass.

### Protein Modeling and Evaluation

We used several protein prediction programs to accurately model tryptogalinin, namely: Scratch Protein Predictor [25], LOOPP [26], Phyre [27], I-TASSER [28], EsyPred3D [29], and Modeller [30]. All predicted models from all programs were then evaluated using the web-server, Qualitative Model Energy ANalysis clustering method (QMEANclust) [31]. QMEANclust allows the user to simultaneously qualify several prediction programs and their generated 3D structures. QMEANclust has a scoring function based on six descriptors, four that are statistical potentials: A geometric potential analyzed by torsion angles; interactions among Cβ atoms and all atoms distance-dependent potentials; and a solvent-based potential for residue burial. The remaining two functions describe the correlation between the predicted and the calculated secondary structure and a solvent accessibility function [31]. The QMEANclust server provides a scoring for the predicted models with a range of 0 to 1 (1 being the best score). Modeller had the best score, and therefore was used hereafter.

### Molecular Dynamics

Molecular dynamics (MD) were performed with Desmond [32]. The structures were solvated in an orthorhombic box, with a buffer solvent region of at least 10Å. The system was neutralized and an ionic force of 0.15M was set. The default relaxation protocol in Desmond was used. The production run was in the NPT ensemble with a Nose-Hoover thermostat and a Martyna-Tobias-Klein barostat. The temperature was set to 300 K with a 2 fs time step, shake on hydrogen atoms and long range Ewald summation.

### Protein-Protein Docking and Structural Refinement

We used several servers to identify a close to native complex between tryptogalinin and trypsin by performing a blind docking (i.e., we did not input any interacting residues between protease and inhibitor) using a trypsin monomer (PDB: 1TLD) [33]. As a true positive control, the same procedure was performed using the monomers for TdPI (PDB: 2UUX), a modeled TdPI (using Modeller) and trypsin (PDB: 1TLD). We found that docking the monomers of TdPI (PDB: 2UUX) and trypsin (PDB: 1TLD) using the ClusPro 2.0 server [34], [35], [36], [37] generated a near to native crystal structure (6.3Å RMSD) compared with other docking programs (>10Å RMSD), such as PyDock [38], [39] and FireDock [40], [41] – data not shown. Normally, poses of ∼10Å RMSD are considered to be a successful docking [39]. Using the modeled TdPI and tryptogalinin, however, generated docked poses >15Å than the native structure and introduced more false positives, even after the refinement methods developed by Masone et al. [42]. We also attempted to indicate specific residues for the ClusPro server that come into contact upon binding (e.g., Lys-Asp), but this did not produce a proper docking pose, increased the number of false positives and reduced the number of generated poses – data not shown. All these shortcomings suggested a robust technique must be applied in our docking methods.

The CG protein-protein docking uses the Basdevant et al. potential [43]. This CG model reduces each residue to one, two or three beads and uses only electrostatic and Van der Waals energy terms. We implemented it on a Monte Carlo search algorithm where, optionally, the search may be biased towards a desired goal by adding geometric constraints. Here, based on the TdPI-trypsin crystal (PDB: 2UUY), we added an 8Å cutoff between Lys13 and Asp191 for tryptogalinin. Starting from a configuration where both monomers are far apart, the algorithm first generates random large configurational jumps (up to 20Å translation and 360° rotation) of the ligand (tryptogalinin) until the distance cutoff is satisfied. Then, the size of the random jumps decrease to perform 10,000 steps of local exploration (up to 3Å and 5°). The overall procedure may be repeated several times. The distance cutoff, together with a steric clash screen, quickly populates the areas of interest (determined by the experimental information, etc.). Furthermore, new configurations are only accepted if five parameters related with relative positions between monomers differ by a range from any previous one. The parameters used to avoid the production of similar results are spherical coordinates of the center of mass of the ligand respect to the receptor and two spherical angles within the ligand. The overall procedure is capable of producing around 300,000 configurations in 10 hours on a single CPU.

All Monte Carlo accepted steps within the cutoff constraint were then clustered to 100 poses and converted back to all-atom models (keeping the initial atomic structure information). Following Masone et al. [42], we refined the all-atom poses using the Schrodinger's Protein Wizard [44] that optimizes the entire hydrogen bond network by means of side chain sampling. The algorithm builds hydrogen-bonded clusters using a criterion of 3.5Å between heavy atoms. The program then performs 100,000 Monte Carlo moves for each cluster reorienting hydroxyl and thiol groups, amide groups of Asn and Gln and the imidazole ring in His. It also predicts the protonation state of His, Asp and Glu. Each possibility is scored based on the quality (from standard geometric parameters) and quantity of hydrogen bonds. The resulting all-atom structures are then minimized with a Truncated Newton algorithm, an OPLS-AA force field, and a surface generalized Born (SGB) implicit solvent.

Binding energies, for both the CG and all-atom potential poses, were estimated by: 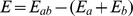, where *E_ab_* is the energy for the complex and *E_a_* and *E_b_* are the energies for their respective monomers.

## Results and Discussion

### *I. scapularis* Monolaris Multiple Sequence Alignment Identifies a Kunitz Peptide with an Unusual Cysteine Motif

**Figure 1:**
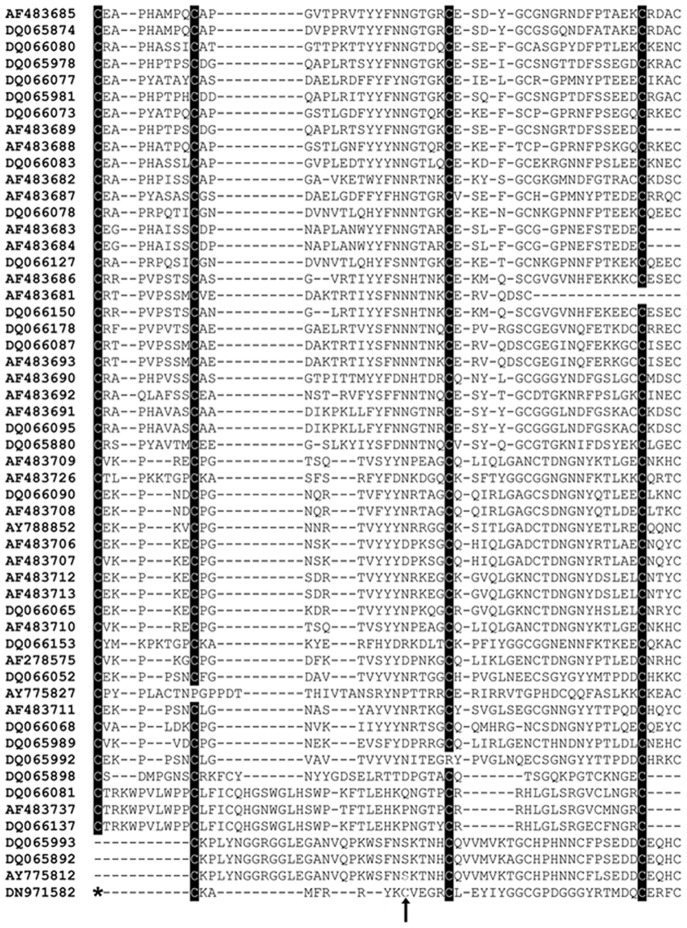
A multiple protein sequence alignment of the monolaris group from the *I. scapularis* sialome project [9]. The multiple sequence alignment is based within the Cys framework showing the conserved Cys residues (black) and the arrow points to the oddly placed Cys position that depicts a potentially ‘atypical’ Cys motif in a monolaris protein described in the sialome of this tick – the asterisk denotes the missing Cys residue. All the monolaris sequences are named after their GenBank accession number and the ‘atypical’ sequence (DN971582) is shown last (at the bottom of the sequence alignment).

Based on our sequence alignment of the conserved Cys residues in Kunitz domain peptides, we identified in the sialome project of Ribeiro et al. [14] a modified Kunitz among all the described *I. scapularis* monolaris sequences. Specifically, one sequence (GenBank: DN971582, the last sequence in Figure 1) is missing the initial conserved Cys residue that forms the first disulfide bridge of archetypical Kunitz peptides (denoted with an asterisk in Figure 1). Figure 1 also shows this peptide possessing an oddly positioned Cys (indicated by an arrow) that does not follow any Kunitz Cys motif found in *I. scapularis* sialome or previously described in the literature [45]. The Cys motif that this Kunitz displays is: CX(8)CX(4)CX(7)CX(12)CX(3)C.

The *I. scapularis* sialome described a few groups of monolaris Kunitz based on their Cys motif that vary according to the number of inter-Cys amino acids (X) [14]. Single domain Kunitz peptides are the largest Kunitz peptides expressed in tick salivary glands and the variations in their Cys motifs have evolved in *I. scapularis* from a single motif known as group 1: CX(8)CX(15)CX(7)CX(12)CX(3)C [45]. Evolutionary expansion of group 1 Kunitz peptides produced a diverse inhibitory effect and/or function increasing the tick’s ability to combat host defense mechanisms (specifically those in the genus *Ixodes*) [45]. Given the divergent nature of tick Kunitz peptides, it is not suprising that variations of the currently known Cys motifs occur. Our study simply adds to the diversity of this specific protein family among the Ixodidae.

### The *I. scapularis* Salivary Kunitz Peptide is a Serine Protease Inhibitor with a Higher Affinity for Human Skinβ-Tryptase

**Figure 2:**
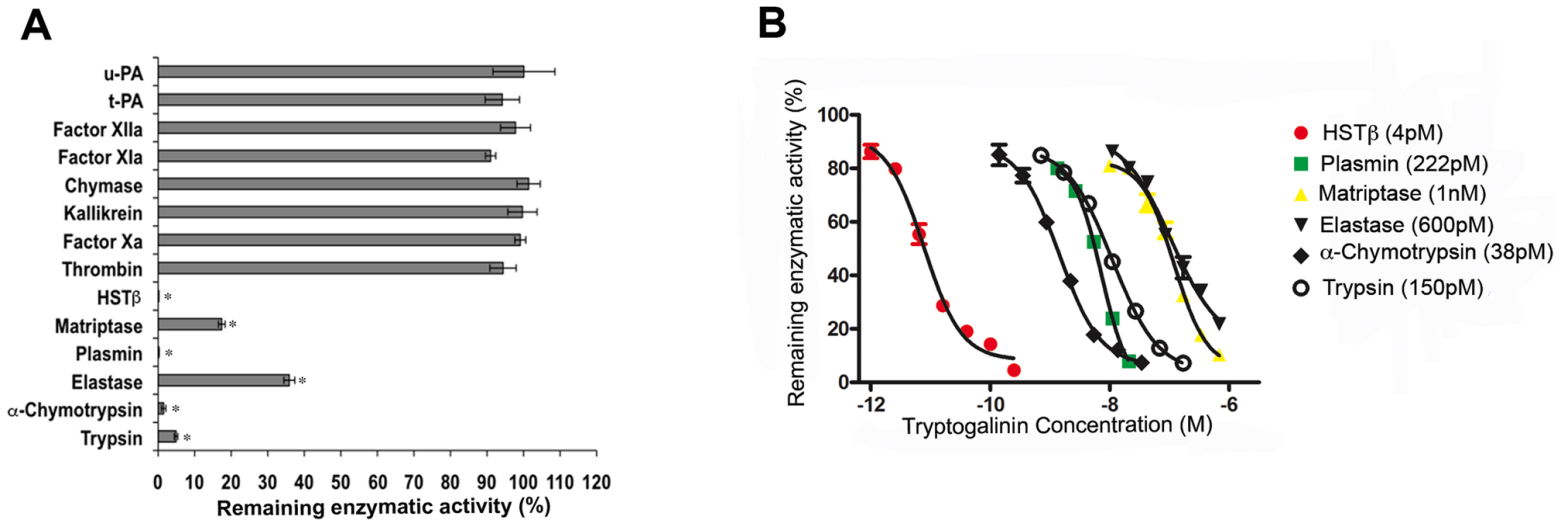
A–B. Titration of the targeted serine proteases with the recombinant protein. An inhibition screening (A) using 14 vertebrate serine proteases – the asterisk denotes with p-values ≤ 0.05 and ≤50% inhibition. Inhibition curves (B) were produced for the targeted enzymes by plotting the concentration of the inhibitor and the estimated percentage of the enzymatic activity. Since the amount of enzyme used in the assay varies for each protease, we represent the molar excess of the *I. scapularis* Kunitz peptide to achieve 50% inhibition of the respective enzyme, when compared to the amount of enzyme used in each assay (also see Materials and Methods).

Compared to vertebrate Kunitz inhibitors that play a role in inflammatory responses, invertebrate Kunitz peptides possess a diverse inhibitory effect on protease activity [46]. To date, hundreds of tick Kunitz peptides have been discovered from sequencing projects [13], [47], [48], [49], [50]; however, as summarized below, roughly a dozen of tick Kunitz peptides are functionally described. Therefore, we overexpressed the divergent salivary Kunitz peptide in order to characterize its potential inhibitory activity against several vertebrate serine proteases. After HPLC purification, the purity of the recombinant peptide exceeded 95% (coomassie stained gel – data not shown). We then tested the recombinant peptide against 14 vertebrate serine proteases. Figure 2A depicts the results showing a statistically significant inhibition for trypsin, α-chymotrypsin, HSTβ, plasmin, matriptase and elastase. Figure 2B shows standard inhibition curves for all the targeted enzymes to further verify that the recombinant protein inhibits the above-mentioned enzymes [51]. Based on the data from these inhibition curves and calculating the *K_m_* of the substrates under the assay conditions (data not shown), we were able to calculate the apparent *K_i_* (*K_i_*_ (app)_) for the targeted enzymes (see Materials and Methods) revealing a low pM *K_i_*_(app)_ for HSTβ (Table 1).

**Table 1:** Protease inhibition by tryptogalinin.

*Enzyme*	*Tryptogalinin K*_i__(app)_ (*nM*)
**Trypsin**	0.49
**α-Chymotrypsin**	0.41
**Elastase**	78.03
**Plasmin**	5.83
**Matriptase**	13.97
**HSTβ**	0.01*
Thrombin	-
Factor Xa	-
Kallikrein	-
Chymase	-
Factor XIa	-
Factor XIIa	-
t-PA	-
u-PA	-

The apparent *K_i_* values (*K_i_*_(app)_) of tryptogalinin for the targeted enzymes were estimated using the BotDB web-server [16].

* Such a calculation is not feasible in the case of HSTβand thus *K_i_*_(app)_ was estimated according to Schaschke et al. [18].

Specific nomenclatures for tick protease inhibitors describe the origin and function of the inhibitor. For example, in the case of sialostatin L [10], [52], [53], [54] ‘sialo’ stands for the salivary origin of the inhibitor and ‘statin’ for its ability to inhibit cathepsin L (the enzyme type is denoted in the name of the inhibitor with the last letter ‘L’). Another two examples denoting the origin of the inhibitor are anophelin (an Anopheles protease inhibitor) [55], [56] and boophilin (Boophilus protease inhibitor) [57]. Since the general term inhibitor does not accurately describe the mechanism or target (i.e., Anophelin and Boophilin mainly inhibit thrombin) we decided that our name synthesis should include the target inhibitor and its function. For this reason we named the protein tryptogalinin: *trypto* from tryptase and *galinin* from the Greek verb ‘galinevo’ meaning to calm down.

Given that the inhibition of HSTβ reached almost 100% (Figure 2A), we concluded that the inhibitor was able to block at least two active centers of the HSTβ multimeric enzyme. Trypsin and α-chymotrypsin were also targeted with high affinity by tryptogalinin (Table 1), but these enzymes were only used in our analysis as archetypes of the trypsin and chymotrypsin-like serine proteases without any physiological relevance in the interaction of tick saliva with the vertebrate host. Another three human serine proteases (plasmin, matriptase, elastase) were also targeted with relatively high affinity (*K_i_*_(app)_ in the low nM range) by tryptogalinin (Table 1), but the calculated *K_i_*_(app)_ for these enzymes were three to four orders greater than that of HSTβ.

**Figure 3:**
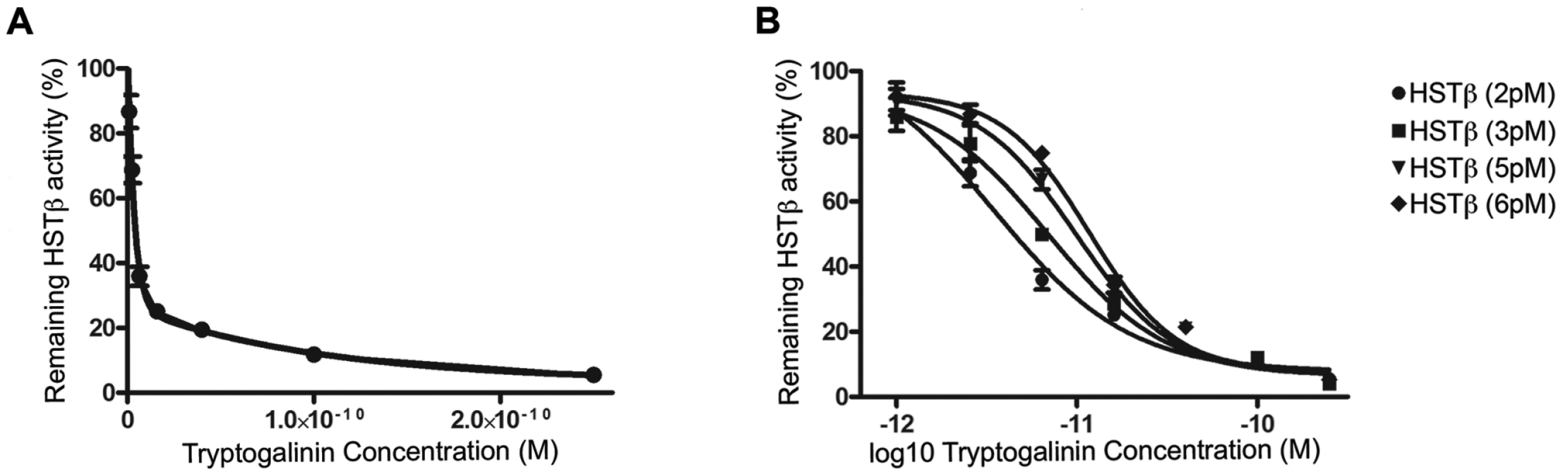
A–B. Inhibition of HSTβ by tryptogalinin. A two-phase exponential fit of the data for HSTβinhibition suggests a 2:1 binding ratio (A) for tryptogalinin and HSTβ (2 pM). Tryptogalinin tightly binds to HSTβ (B) – as the enzyme increases so does the *IC50*.

In Figure 3A we show that inhibition of HSTβ by tryptogalinin gave a two phase exponential fit indicating that tryptogalinin binds to HSTβ in 2:1 ratio (inhibitor:enzyme); this is further supported with the fact that we used 9.6 pM of tryptogalinin for a 50% inhibition of 5 pM of HSTβ (*IC50*). Figure 3B describes tryptogalinin as a tight binding inhibitor of HSTβ since the inhibition curves were different when we increased the amount of enzyme used in our assays. Finally, we show that there is a direct correlation between the amount of HSTβ used in the assay with the estimated *IC50* (see Figure S1) – a typical characteristic of tight binding inhibitors [52]. The HSTβis a trypsin-like protease found in mast cells and a key player in inflammatory responses [58], therefore, our current study may prove useful for any future pharmaceutical studies. It is worth noting that the specificity of β-tryptase function and inhibition is due to its tetrameric structure [59]. To date, there are only two resolved crystal structures that are potent inhibitors of β-tryptase: TdPI and leech-derived tryptase inhibitor [60], [61].

### Evolutionary Relationship of Tryptogalinin with Other Functionally Described Kunitz Protease Inhibitors

**Figure 4:**
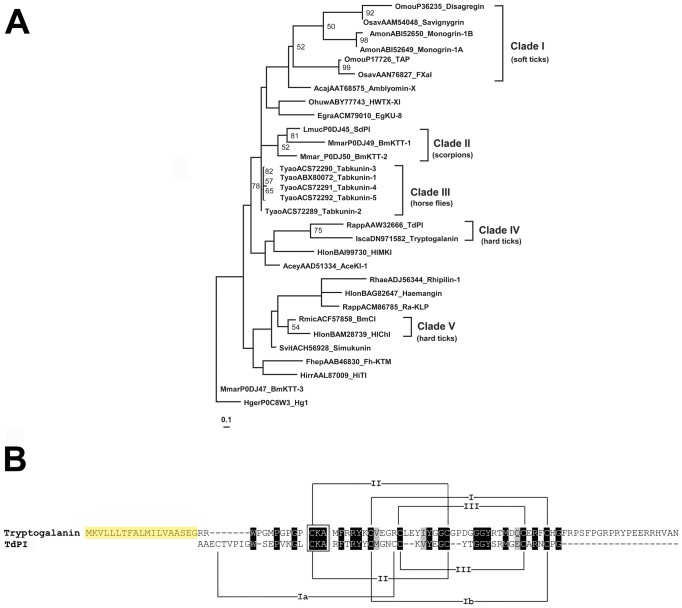
A–B. Phylogenetic reconstruction and sequence analysis of tryptogalinin and functionally described Kunitz from the literature. A phylogram (A) of tryptogalinin and other functionally described Kunitz peptides from hematophagous arthropods, nematodes and platyhelminthes was constructed using maximum likelihod (ML) methods. The first four letters for each label displays the taxa name followed by the GenBank accession number and the functional nomenclature from the literature. The presented ML tree was rooted with Hg1, a trypsin and potassium channel inhibitor of the Mexican scorpion *Hadrurus gertschi* (GenBank: P0C8W3). Bootstrap values (≥50%) are indicated in the phylogram and the scale bar (mean amino acid substitution/site) is presented at the bottom left corner. A sequence alignment (B) of tryptogalinin and TdPI of *Rhipicephalus appendiculatus* from clade IV. Identical (black) and similar sequence regions (grey), the positions of the conserved Cys residues (roman numerals and brackets), and the Cys-Lys-Ala sequence (box) that form the Kunitz head (P1) interacting with the active site of serine proteases are marked. The signal peptide for tryptogalinin is highlighted in yellow.

Since we showed that tryptogalanin inhibits several serine proteases, we were interested in the relationship of this protease inhibitor to other functionally described Kunitz peptides from hematophagous arthropods, nematodes and platyhelminthes. Our analysis showed that the overall phylogenetic relationship among these Kunitz peptides was not resolved using maximum likelihood (ML) methods, apparently due to their amino acid sequence diversity. Only a few internal clades in the ML tree showed bootstrap support values higher than 50%. The phylogram in Figure 4A reveals five well-supported clades of Kunitz protease inhibitors in soft and hard ticks, scorpions and horse flies.

The best-supported group in the phylogram of Figure 4A was clade I that included inhibitors from the soft tick genera *Ornithodoros* and *Argas*. These Kunitz peptides possess a 6-Cys framework with 5 or 6 amino acid residues between Cys residues 3 and 4 in comparison with all other Kunitz peptides (see Figure S2). The monogrins of *A. monolakensis* (1A and 1B) [62] are orthologs of Disagregin [63] and Savignygrin from *Ornithodoros* spp. [64]. These all share the RGD integrin recognition motif with the exception of Disagregin that possesses a glutamic acid instead of a glycine. All these peptides inhibit platelet aggregation. The orthologs TAP of *O. moubata* [4] and FXaI of *O*. *savignyi* [65] share 47% sequence identity and both peptides are FXa inhibitors of the blood-clotting cascade.

Clade II in Figure 4A was the second best-supported Kunitz group that consisted of only scorpion venom peptides. These peptides present a unique Cys framework of either 6 or 8 Cys residues compared with other Kunitz peptides and they are all potent trypsin inhibitors that share a double Cys at the C-terminus (Figure S2). Analysis of the tertiary structure demonstrated that, compared with classical Kunitz, SdPI apparently adapts a new disulfide bridge at the C-terminus and lacks the archetypical Cys 2 and Cys 4 disulfide bridge [66]. The interaction site of SdPI at the N-terminus with trypsin, however, did not change due to this structural deviation. BmKTT-1 shows a 56% protein sequence identity with SdPI and thus demonstrates an ortholog protein form [24]. BmKTT-1 and BmKTT-2 are bifunctional toxins that block potassium channels – in addition to their trypsin inhibitory activity [24]. The same functional characteristics were discovered for the scorpion venoms BmKTT-3 and Hg1 [24], and the spider venom HWTX-XI [67] that are clearly not resolved in the phylogram (apart from Hg1 used as an outgroup; Figure 4A). Additionally, Hg1 demonstrated to be a specific Kv1.3 channel blocker with a channel interaction site at the C-terminus instead of the N-terminal region in classical Kunitz peptides.

Clade III contained Kunitz peptides from the horse fly *Tabanus yao* (Figure 4A). As Figure S2 depicts, these so called tabkunins (1–5) possess archetypical Kunitz domains of a 6-Cys framework [68], [69] that is common for other serine protease inhibitors – mainly from ticks (e.g., HlonBAI99730_HlMKI, RmicACF57858_BmCI and HlonBAM28739_HlChI, in the phylogram), Diptera (e.g., SvittACH56928_Simukunin and HirrAAL87009_HiTI, in the ML tree) and platyhelminthes (e.g. FhepAAB46830_Fh-KTM and EgraCM79010_EgKU-8, in the phylogram). All tabkunins possess anti-coagulation properties via inhibition of thrombin and are also inhibitors of trypsin, elastase and chymotrypsin [68], [69].

Figure 4A also shows two additional clades (IV and V) of hard tick Kunitz peptides that are well supported in the ML tree. Clade V contained BMCL of *Rhipicephalus microplus* [70] and HlChl of *Haemaphysalis longicornis* [71]. Both Kunitz peptides compared with all other functionally described peptides possess a tyrosine at the P1 position of the inhibitor interactive site suggesting a chymotrypsin inhibitory activity [70], [71] (see Figure S2). Both protease inhibitors are extremely selective for chymotrypsin. Additionally, BMCl is a dendrotoxin-like protein that is able to induce apoptosis [70].

In Figure 4A, clade IV presents tryptogalinin together with TdPI, another potent human β-tryptase inhibitor from the hard tick *Rhipicephalus appendiculatus* [5]. Both peptides possess the same Cys-Lys-Ala (C-K-A) motif that form the enzyme-inhibitor interactive site (P1) and a slightly shifted Cys framework compared to the other Kunitz peptides from the phylogram (see Figure 4B and Figure S2). TdPI has an overall altered Kunitz domain structure due to a lack of an alpha-helix, shortening of a loop region, differences in disulfide-bridges (four as opposed to three found in classical Kunitz), and a relocation of the N-terminus [5]. Structural differences, compared with classical Kunitz peptides, in the loop regions of TdPI generate an “arrow-like” structure that increases TdPI association with the compact binding site of trypsin and β-tryptase. We used the web-server DiANNA [72] to predict the disulfide bridges – also verified by our homology modeling (see below) – demonstrating that both TdPI and tryptogalinin share similar disulfide bridges (except for the additional Ia disulfide bridge in TdPI, Figure 4B). As most Kunitz protease inhibitors, but unlike TdPI, tryptogalinin possesses six Cys residues forming three disulfide bridges. The orders of the disulfide bridges, however, differ from that of classical Kunitz proteins since they form a pattern similar to TdPI – the first disulfide bridge is in the same conformation as the Ib disulfide bridge of TdPI (Figure 4B).

Although TdPI and tryptogalinin derive from two completely different tick genera located in separately distinct geographical regions, these two hard ticks possess a salivary protease inhibitor with similar protease inhibitory targets. Compared with TdPI, however, tryptogalinin shows a broader spectrum (and greater affinity) against additional serine proteases that play a role in inflammation and vertebrate immunity [58], [73], [74], [75].

### Tryptogalinin Homology Model and its Structural Relation to the Kunitz Family

Two naturally evolved proteins with >25% identical residues are extremely likely to be similar in their tertiary structure [76]. Therefore, due to the sequence similarity and phylogenetic relationship between tryptogalinin and TdPI, we ultimately used homology modeling methods to predict its overall structure. To achieve the best possible tertiary model for tryptogalinin, however, we incorporated several protein prediction programs and evaluated the output structures using QMEANclust [31]. Modeller [30], [77] outranked the other prediction programs with a QMEANclust score of 0.82 (see Materials and Methods for QMEANclust scoring function). For Modeller, we used the crystal structure of TdPI (PDB: 2UUX) as a template to model tryptogalinin (i.e., homology modeling).

**Figure 5:**
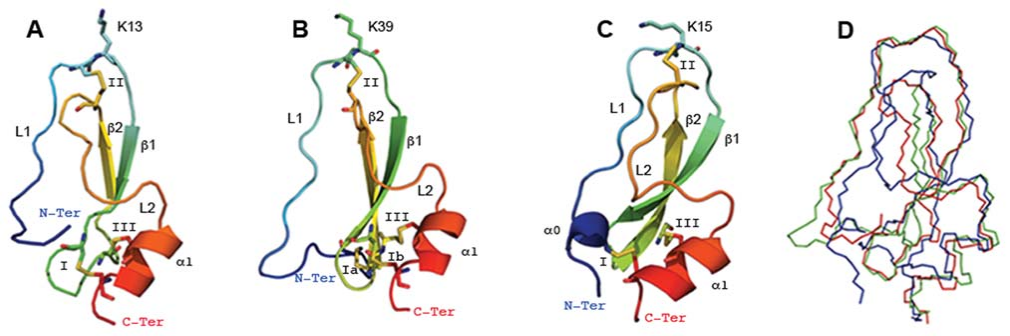
A–D. Tryptogalinin predicted tertiary structure. The modeled tryptogalinin (A), TdPI (PDB: 2UUX) (B) and BPTI (PDB: 5PTI) (C) depict the conserved disulfide bridges (indicated by roman numerals), loops (L1 and L2), the beta-sheets (β1–β2) that form the b-hairpin, the alpha-helixes (α0 and/or α1) and the Lys (K) that interacts with the active site of serine proteases. The Cα superimposition (D) of tryptogalinin (red), TdPI (green) and BPTI (blue) show the similarities/differences in their overall structures.

The tertiary homology structure of tryptogalinin resembles that of TdPI since it contains a short α-helix (α1; seven residues) and lacks the N-terminus 3_10_ α-helix, α0 (Figure 5A-B). The N-terminus α0 is usually a common motif found among Kunitz peptides (see BPTI in Figure 5C). Tryptogalinin also possess the archetypical anti-parallel β-sheets, but the β-hairpin is longer in tryptogalinin (twelve residues) compared to TdPI (two residues) and when compared with the archetypical Kunitz (four to six residues); however, this may be due to the shorter β-sheets of tryptogalinin. It is worth noting that secondary structures do not drastically change throughout evolution (e.g., insertions/deletions) and a common obstacle for 3D modeling programs is to accurately predict β-sheet conformations [76]. We attempted to perform evolutionary protein model building by using Phyre2 [27]. Although Phyre2 provided a 3D model accurately predicting tryptogalinin’s β-sheets (7 aa as opposed to the 4 aa predicted by Modeller), the models produced had low QMEAN score, a truncation at both termini, and the disulfide bridges were not well organized thereby reducing the number of bridges (2 instead of 3) – data not shown.

**Figure 6:**
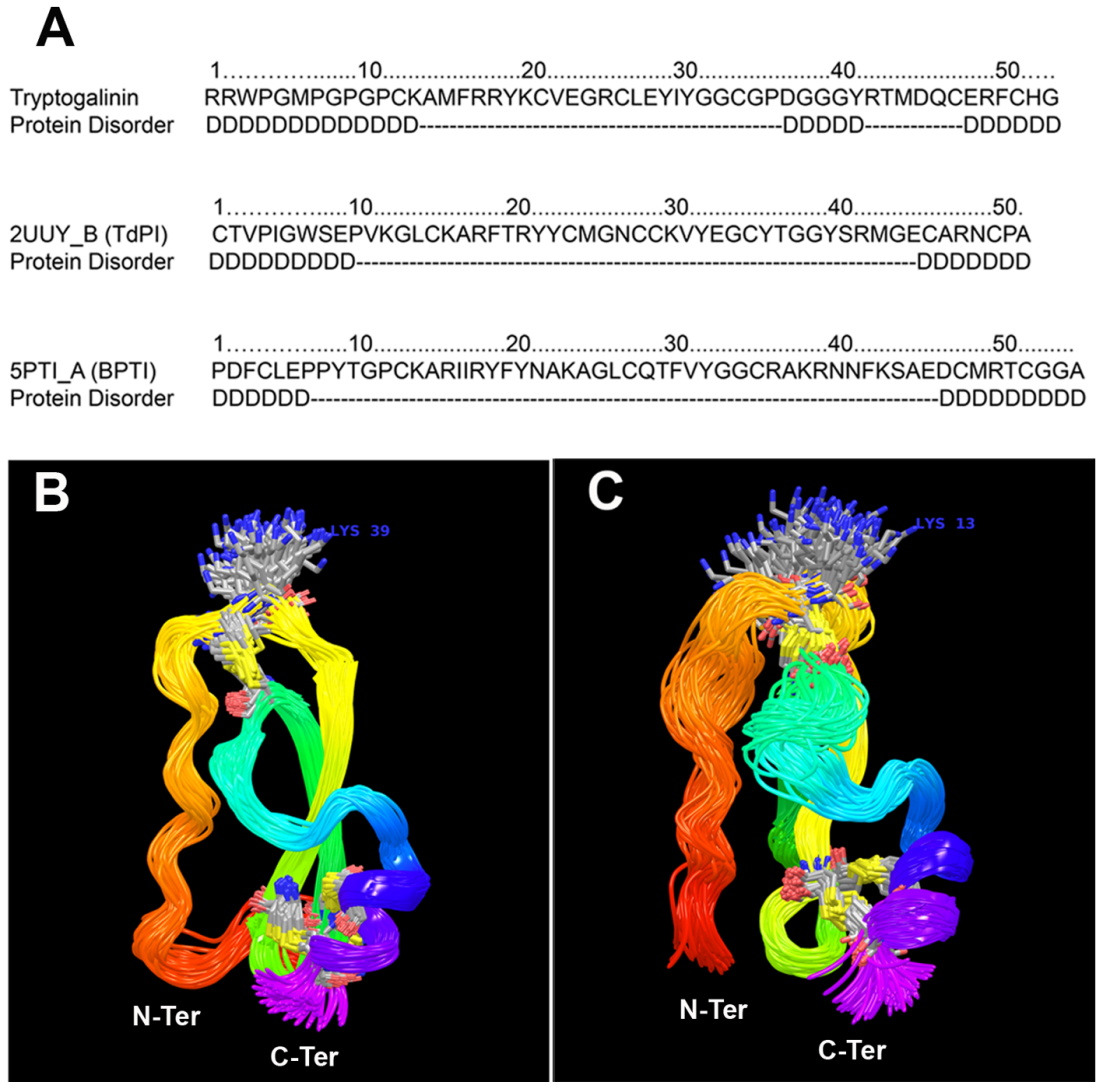
A–C. Intrinsic protein disorder and MD simulations. Intrinsic regional protein disorder (A) predicted by the GeneSilico MetaServer [37] for tryptogalinin, TdPI (PDB: 2UUY chain: B) and BPTI (PDB: 5PTI), respectively – ‘D’ represents the disordered region and dashes are ordered. A total of 100 snapshots (i.e., conformations) during last 40 ns of MD for TdPI (B) and tryptogalinin (C).

The loop region L2 of tryptogalinin is similar to classical Kunitz peptides (eleven residues, as in BPTI tharegional protein disorder (A) predictedt has twelve) distinguishing tryptogalinin from TdPI (with only eight residues). Both L1 and L2 are thIntrinsic regional protein disordere main determinants on forming the Kunitz head that interacts with the active site of serine proteases [5]. Another main characteristic that distinguishes tryptogalinin from the majority of Kunitz peptides (including TdPI) is that the N-terminus (L1) is detached from the rest of the peptide due its lack of the first disulfide bridge, a unique structural distinction between the two peptides. This regional difference also translates into a high regional disorder as predicted by the MetaDisorder server [78] compared with TdPI and BPTI (Figure 6A). (MetaDisorder uses 12 separate programs and builds a consensus to predict protein disorder.)

### Molecular Dynamics and Intrinsic Protein Disorder Reveal Tryptogalinin’s Biochemical Interactions

Tryptogalinin is an excellent candidate for refinement techniques using molecular dynamics (MD) due to its small size and the presence of multiple Cys bridges; therefore, we refined the homologous tryptogalinin model with a 60 ns trajectory. As expected from a homology-modeled structure, we observed a rapid deviation from the initial conformation (∼4Å RMSD; Figure S3), followed by an equilibration. Figure 6B shows 100 equidistant structures for the last 40 ns and compares them to a TdPI simulation (Figure 6C; under the same conditions). In agreement with our primary sequence analysis (i.e., intrinsic regional disorder – Figure 6A), we observe larger mobility in the L1 (as a result of the missing N-terminus disulfide bridge) and the L2 loop regions for tryptogalinin. Furthermore, this higher regional mobility results in the lysine 13 (Lys13) residue to explore a significantly larger area of space.

Intrinsically disordered regions increase molecular recognition because of an ability to fold differently upon binding as well as possessing large interacting surfaces [79]. This may explain tryptogalinin’s high affinity and multiple serine protease inhibition since part of its disorder extends from the N-terminus (L1) to the P1 interacting site (K13) compared with TdPI (Figure 6A). Disorder is also predicted in the L2 region in proximity to the fourth Cys residue (the Cys forming the disulfide bond II with the P1 site). Such mobility, however, might result into an induced fit recognition mechanism, therefore complicating any protein-protein docking simulations.

### Tryptogalinin-Trypsin Docking Verifies an Induced Fit Recognition Mechanism

Since the TdPI-trypsin crystallographic structure has been solved (PDB: 2UUY), we attempted to predict the tryptogalinin-trypsin complex by performing protein-protein docking. Initial blind docking of the homology model and of the last structure from the equilibration MD with ClusPro 2.0 [34], [35], [36], [37], PyDock [38], [39] and FireDock, [40], [41] did not produce any result <10Å RMSD; the best scoring poses were located at RMSD distances >20Å. Inspecting the generated poses it was apparent that Lys13 (and L1) was not able to approximate towards the trypsin active binding site. The distance between Lys13 of tryptogalinin and the Asp191 of the trypsin binding site was always >8Å, whereas the distance between TdPI Lys39 and trypsin Asp191 is ∼3Å in the 2UUY crystallographic structure. Furthermore, when trying to superimpose the tryptogalinin model (or the MD equilibrated one) to TdPI in the 2UUY crystal, it was clear that the Lys conformation was significantly different from the one present in the TdPI crystal. Together with the MD results shown above, all these data point to a possible induced fit or a conformational selection mechanism for tryptogalinin.

**Figure 7:**
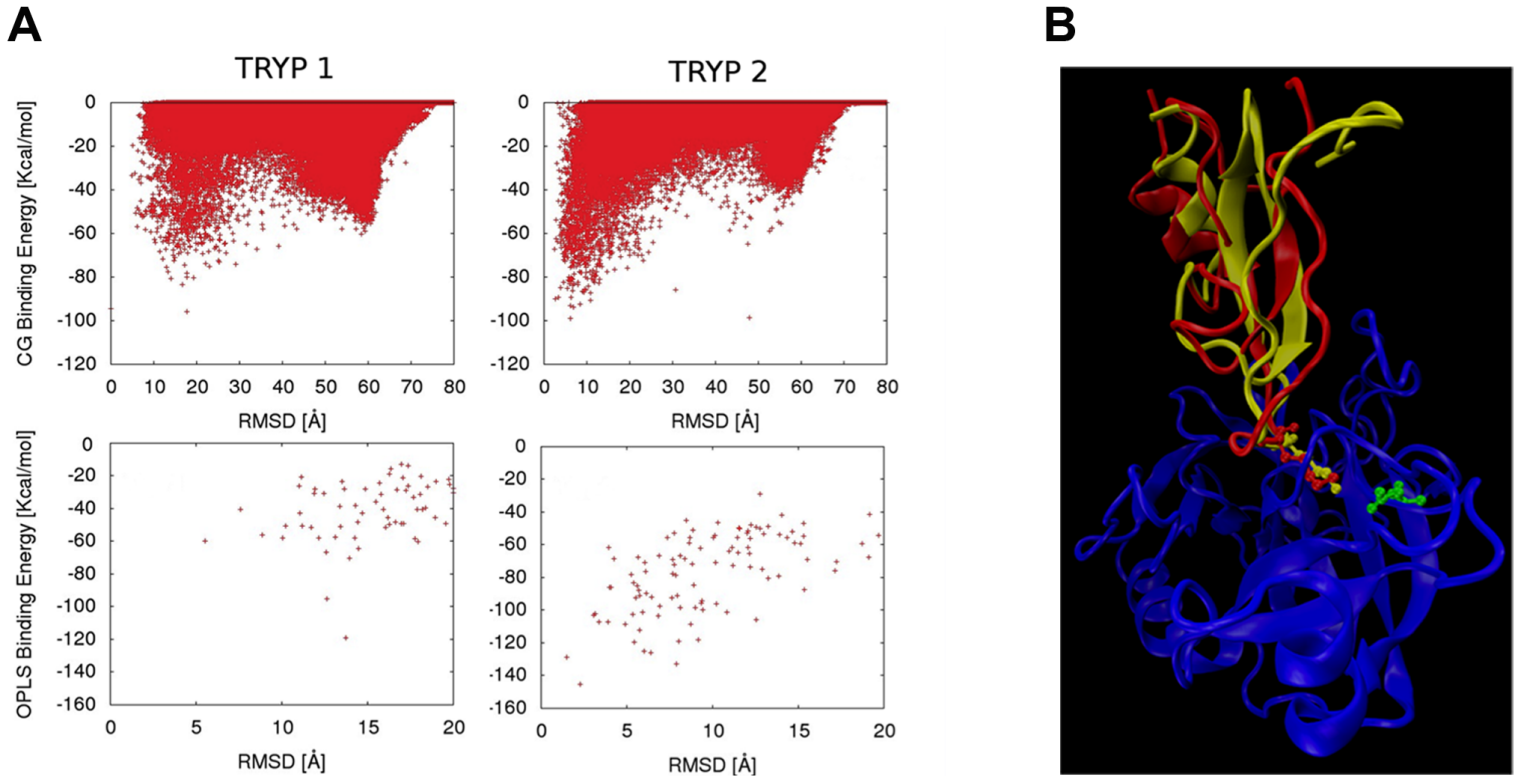
A–B. Coarse grain docking of refined tryptogalinin model and tryptogalinin-trypsin complex. Top two panels (A) show the coarse grain binding against the RMSD for two tryptogalinin models, Tryp1: the last snapshot of a 62 ns equilibration, and Tryp2, the snapshot with best superimposition to TdPI (its complex with trypsin) (PDB: 2UUY). RMSD were obtained with respect to the superimposition of tryptogalinin to 2UUY. Bottom panels show the all-atom binding energy after clusterization of the coarse grain poses. A comparison between (B) the best all-atom model for tryptogalinin (yellow) and the complex TdPI-trypsin crystal structure (PDB: 2UUY) (red) depict significant binding similarities. Lys13 and 34, and Asp191 (green) are represented in ball and stick.

To further test this hypothesis we proceeded by superimposing all the tryptogalinin MD snapshots to TdPI and found one structure with only 0.9Å RMSD (for the Lys all-atom RMSD). Then we used this structure (Tryp2), plus the equilibrated MD model (Tryp1; with a ∼4Å Lys-Asp RMSD), for the following round of protein-protein docking studies. This time, we used a biased docking approach based on the Basdevant et al. [43] coarse grain (CG) potential, a model with only 2–3 beads per residue that softens the steric repulsion and favors the surface contacts. The top two panels in Figure 7A shows 300,000 Monte Carlo steps for the CG exploration, where we biased tryptogalinin to the active site (see Materials and Methods section). Tryp2, the tryptogalinin MD conformation with better superimposition to TdPI, enters the active site reaching Lys13-Asp191 distances <4Å with a significant correlation between the CG binding energy and the RMSD. In agreement with the previous docking experiments, the equilibrated MD structure is not capable of entering the active site. From these CG results we clustered 100 poses where we imposed the distance between Lys13 and Asp191 to be <8Å, and refined them with all-atom models (see Materials and Methods section). The bottom two panels in Figure 7A show the all-atom binding energy. Clearly, Tryp2 produces again a significant correlation of the binding energy with the RMSD, using the 2UUY model as reference, indicating that both proteins bind similarly (as could be expected by their phylogenetic relation and similarities in protease inhibition – e.g., β-tryptase).

Figure 7B shows a comparison between the TdPI crystallographic structure and the best tryptogalinin model after the all-atom refinement. The Lys orientation, α-helix and β-sheet placement significantly agree with that of the TdPI crystal complex. We should emphasize here that the only constraint in the simulation was the Lys-Asp distance, maintaining it below 8Å (with a crystal value of 3.4Å). Interestingly, the presence of a conformation in the tryptogalinin dynamics matching the TdPI bound structure, and producing similar bound complex with the best scoring, indicates a conformational selection binding mechanism.

## Conclusion

By combining computational and experimental methods we were able to functionally characterize a single Kunitz peptide (tryptogalinin) from *I. scapularis* that displays modified target specificity when compared with another functionally characterized Kunitz peptide, TdPI. Regardless that these two peptides are secreted from ticks of two separate genera and geographically distinct regions, tryptogalinin and TdPI are closely related when phylogenetically compared with several functionally described Kunitz peptides from the Acari subclass. We show that tryptogalinin inhibits several serine proteases involved in inflammation and vertebrate immunity [58], [73], [74], [75], which may facilitate tick blood feeding. Tryptogalinin has an atypical N-terminus compared with previously described Kunitz peptides that is also highly disordered. We hypothesize that the inhibitory profile of tryptogalinin is due to its intrinsic regional disorder, clearly shown in our molecular dynamics simulations. Conventional docking methods proved to be inadequate due to the conformational selection binding mechanism of tryptogalinin. A theoretical combination of molecular dynamics, superimposition to the TdPI crystal, coarse grain Monte Carlo protein-protein docking, and all-atom refinement procedure, provided an adequate tryptogalinin-trypsin complex.

Our current findings add to the understanding of the molecular evolution of Kunitz peptides in ticks; more specifically, we show that the tick *I. scapularis* has acquired in its salivary secretion a protein with a rather modified Kunitz-fold. The sequence and folding divergence of tryptogalinin allowed the protein to retain its function as an HSTβinhibitor, while possessing an intrinsic regional disorder when compared to TdPI – another HSTβinhibitor of the Kunitz family from the tick *Rhipicephalus appendiculatus*. Accordingly, this is another example of the evolutionary pressure that ticks are subjected to due to their continuous contact with the host (i.e., although tryptogalinin has retained its high affinity to HSTβ,it has acquired some additional molecular targets). Understanding this diversity on how different tick species adapt their salivary secretion to obtain a successful blood meal may lead to discovering and/or engineering highly specific pharmacological agents. Equally important is the valuable insight of the driving forces in the molecular evolution of major protein families (e.g., members of the Kunitz family).

## Supporting Information

Figure S1 **Verifying inhibition of HSTβby tryptogalinin.** There is a strong linear correlation between the amount of HSTβ used in the assays and the observed *IC50* of tryptogalinin (*R^2^* = 0.97).
(TIF)

Figure S2 **Alignment of tryptogalinin and functionally described Kunitz peptides from hematophagous arthropods, nematodes and platyhelminthes.** Mature amino acid sequences of all peptides were aligned by a homology alignment profiling strategy using the program MAFFT version 7 with an iterative refinement method (L-INS-I) and the BLOSUM 62 matrix (Gap opening penalty: 1.5, Offset value: 0.1) [20]. The first four letters of each protein label display the taxa name followed by the GenBank accession number and the functional nomenclature from the literature.
(TIF)

Figure S3 **MD simulation of the modeled tryptogalinin.** The chart represents the Cα RMSD (Å) of tryptogalinin (compared with its native orientation) after a 62.2 ns MD simulation. There were a total of 12961 frames and each frame was saved every 4.8 ps.
(TIF)
