# Supplementary figures and images for: Tryptogalinin Is a Tick Kunitz Serine Protease Inhibitor with a Unique Intrinsic Disorder
Source: PLoS One. 2013 May 3;8(5):e62562. doi: 10.1371/journal.pone.0062562 (PMC3643938; doi:10.1371/journal.pone.0062562)

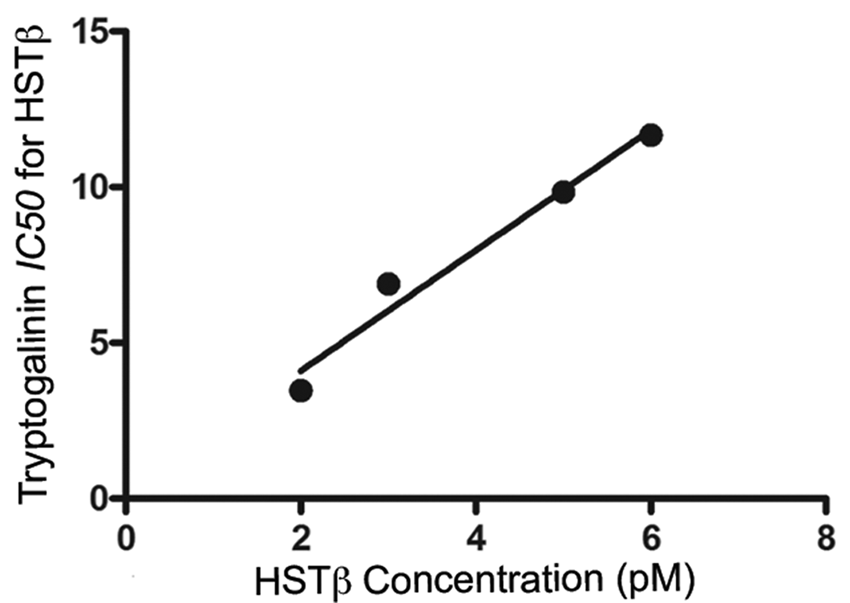

Supplement: Figure S1 — Verifying inhibition of HSTβby tryptogalinin. There is a strong linear correlation between the amount of HSTβ used in the assays and the observed IC50 of tryptogalinin (R2 = 0.97). (TIF) [file pone.0062562.s001.tif]

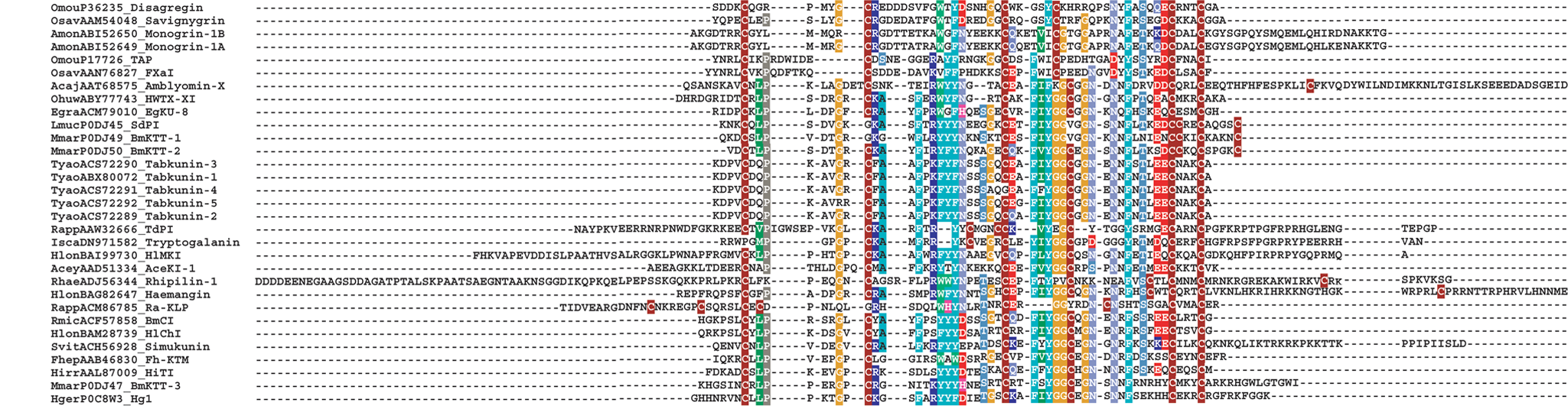

Supplement: Figure S2 — Alignment of tryptogalinin and functionally described Kunitz peptides from hematophagous arthropods, nematodes and platyhelminthes. Mature amino acid sequences of all peptides were aligned by a homology alignment profiling strategy using the program MAFFT version 7 with an iterative refinement method (L-INS-I) and the BLOSUM 62 matrix (Gap opening penalty: 1.5, Offset value: 0.1) [20]. The first four letters of each protein label display the taxa name followed by the GenBank accession number and the functional nomenclature from the literature. (TIF) [file pone.0062562.s002.tif]

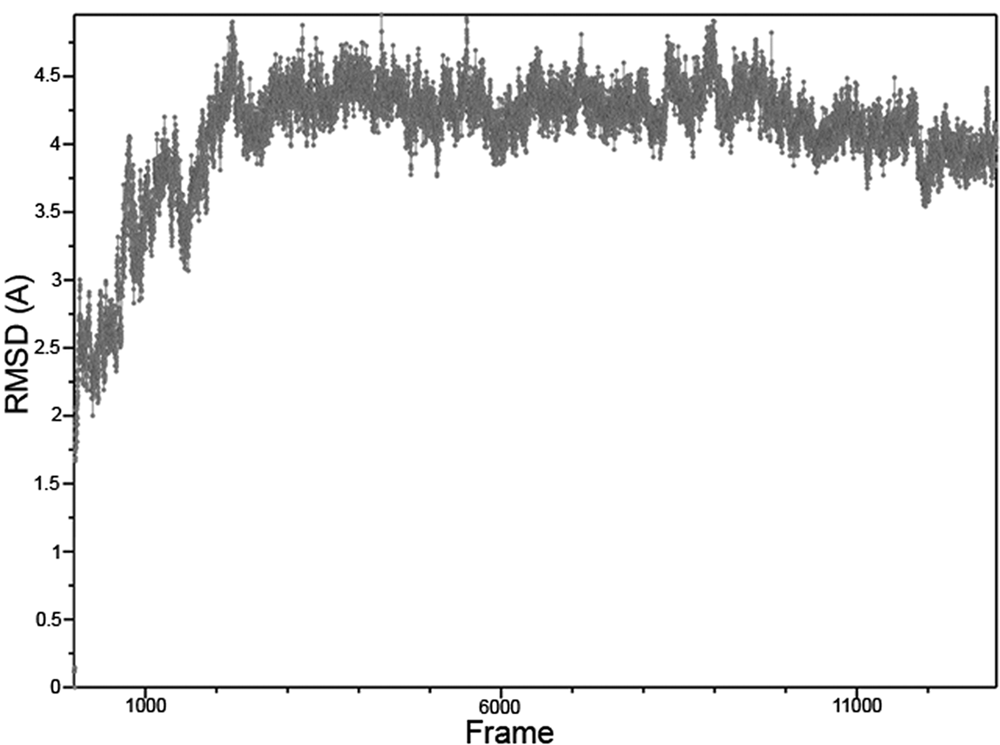

Supplement: Figure S3 — MD simulation of the modeled tryptogalinin. The chart represents the Cα RMSD (Å) of tryptogalinin (compared with its native orientation) after a 62.2 ns MD simulation. There were a total of 12961 frames and each frame was saved every 4.8 ps. (TIF) [file pone.0062562.s003.tif]
